# Retinal vasculitis in HLA-A29 birdshot retinochoroiditis, particularities and imaging narrative of an under-estimated and diagnostic component of the disease

**DOI:** 10.1186/s12348-024-00406-0

**Published:** 2024-07-09

**Authors:** Jérôme Galand, Ioannis Papasavvas, Carl P. Herbort

**Affiliations:** 1 Inflammatory and Retinal Diseases, Centre for Ophthalmic Specialised care (COS), 6, Rue Charles-Monnard, Lausanne, 1003 Switzerland; 2 Swiss Visio and Montchoisi Eye Centre, Lausanne, Switzerland; 3 Uveitis Department, Moorfields Eye Hospital, London, UK

**Keywords:** Birdshot retinochoroiditis, Retinal vasculitis, HLA-A29 MHC antigen

## Abstract

**Background:**

HLA-A29 birdshot retinochoroiditis (BRC) is a primary stromal choroiditis (PSC), the hallmark being the choroidal rice-shaped hypopigmented fundus lesions (“birdshot lesions”). BRC is characterised by dual independent retinal vasculitis and choroiditis, the former often preceding manifest choroidal lesions. The purpose of this study was to analyse the type and severity of retinal vasculitis and determine whether it represented a diagnostic contribution. Medical records of patients with the diagnosis of BRC examined in the uveitis clinic of the Centre for Ophthalmic Specialised care (COS) in Lausanne from 1994 to 2020, were retrospectively reviewed. All patients had a complete ophthalmic examination, including visual field testing, optical coherence tomography (OCT), and fluorescein (FA) and indocyanine green (ICGA) angiography. Key retinal angiographic features were assessed. The study also established the angiographic score for retinal (FA) compared to choroidal involvement (ICGA). Among the 2102 newly diagnosed patients, 33 (1.57%) were diagnosed as BRC. Of the 21 patients with sufficient data included, all exhibited bilateral retinal vasculitis, of which 5 (24%) had no “birdshot lesions” at presentation with ICGA however always showing choroidal involvement. FA characteristics included (1) profuse retinal exudation in 17/21 cases (81%), (2) macular oedema in 17 patients (81%) with foveolar sparing for 14 of them (82%), (3) thick sheathing/staining of large posterior pole vessels in 13 patients (62%) and (4) profuse disc hyperfluorescence in all 21 patients. (5) A specific feature was the so-called pseudo arterio-venous circulatory delay in 17/21 cases (81%). The FA angiographic score at presentation was 14.49 ± 5.1 equivalent to the ICGA angiographic score of 14.29 ± 3.6, and higher than in other chorioretinitis entities. Both angiographic scores decreased similarly after treatment with a slower response of the retinal involvement.

**Conclusions:**

Retinal vasculitis in BRC is often very pronounced and presents distinct angiographic features that help substantially in the diagnosis and understanding of the disease course. Retinal vasculitis can present initially as an isolated feature in absence of the characteristic “birdshot lesions”. The presence of all or some of the specific FA features strongly orient towards BRC to seek confirmation by ICGA and the search for the HLA-A29 antigen.

## Introduction

HLA-A29 birdshot retinochoroiditis (BRC) is a predominantly posterior uveitis involving both eyes and resulting in concomitant but independent retinal vasculitis/retinitis and choroiditis [1]. Its diagnosis, treatment and follow-up require an understanding of both aspects. The disease has been identified, since the early descriptions, with the rice-shaped cream coloured depigmented fundus “birdshot lesions”, considered as the hallmark of the disease [2]. Thus, the choroidal involvement represented the principal focus of attention for many years. Indeed, when indocyanine green angiography (ICGA) became available, the precision of choroidal investigation increased substantially for both diagnosis and follow-up of the disease, becoming a key element in the early diagnosis and its general appraisal [3]. The ICGA aspect has been described thoroughly in multiple articles since the late-1990s [4, 5] with an accent on the hypofluorescent dark dots (HDDs) [6]. The crucial importance taken by ICGA resulted in a continued disinterest of the retinal involvement. The retinal component of the disease has thus often been somewhat neglected.

However, the analysis and characterisation of retinal vasculitis, an early manifestation of the disease, contributes substantially to a better understanding of the disease and a more global appraisal. Logically, retinal involvement was first described by Ryan and Maumenee, Kaplan and Aaberg followed by Gass, between 1980 and 1981, as only fluorescein angiography (FA) was available then [7–9]. Gass mentioned a vasculitis of both small and large vessels. He also mentioned delayed staining of large veins which he falsely interpreted as a real arterio-venous perfusion delay, which was shown in fact to be a pseudo-delay [9, 10]. Inflammation at the retinal and posterior vitreous level is thought to cause most of the symptoms and morbidity such as visual field impairment and floaters [11]. The former is attributed to the very leaky vasculitis causing macular oedema and the latter to posterior vitritis causing myodesopsias [12]. Optical coherence tomography (OCT) is a more recent useful non-invasive modality to analyse the retina, showing that retinal morphology evolves during the course of the disease, beginning with a thickening in the acute exudative phase and becoming progressively thinner with chronicity [13]. Retinal status and evolution are, however, mainly investigated by FA. Other functional and morphological investigations useful to monitor retinal involvement include visual field testing (VF), microperimetry (MP) and full-field electroretinography and more recently OCT angiography (OCT-A) [14–16].

The scope of this work was to systematically analyse the retinal involvement in BRC by precisely and comprehensively pointing out the particularities of BRC related retinal vasculitis and determine its diagnostic potential towards early diagnosis.

## Patients and methods

We retrospectively reviewed charts of patients with the diagnosis of HLA-A29 birdshot retinochoroiditis (BRC) seen in the uveitis clinic of the Centre for Ophthalmic Specialised care (COS) in Lausanne from 1994 to 2020. Patients with sufficient data were retained for analysis.

A complete work-up was performedn all patients including Snellen visual acuity (VA), slit-lamp examination, applanation tonometry, dilated fundus examination, laser flare photometry, computerised visual field with Octopus 900 (Haag-Streit, Köniz, Switzerland), macular optical coherence tomography (OCT) with Heidelberg Spectralis (Heidelberg Engineering, Heidelberg, Germany), and angiography (fluorescein and indocyanine green) (Heidelberg Spectralis). We excluded patients with insufficient data and follow-up.

We studied key features of retinal inflammation using multimodal imaging and functional investigations, in particular using an angiographic scoring method that has been previously described [17].

We also reviewed the literature regarding retinal involvement in BRC.

In early and asymmetrical cases with visual field changes, standard treatment typically involved sub-Tenon’s triamcinolone acetonide (Kenacort®) injections. In situations of insufficient response or symmetrical bilateral involvement, corticosteroids were combined with one or two immunosuppressants. This work does not aim to explore treatment, and details will not be provided.

## Results

### Demographics

Among the 2102 newly diagnosed patients from 1994 to 2020 in our centre, 33 patients (1.57%) were diagnosed with BRC. The mean age of patients was 52.4 ± 9.7 years, including 23 females and 12 males (65% / 35%). HLA-A29 antigen was present in all patients (100%). Of these patients, 21 out of 33 were seen since the initial onset of the disease and had sufficient data and follow-up information to be included in the analysis.

### Fluorescein angiography (FA) findings

**Fig. 1:**
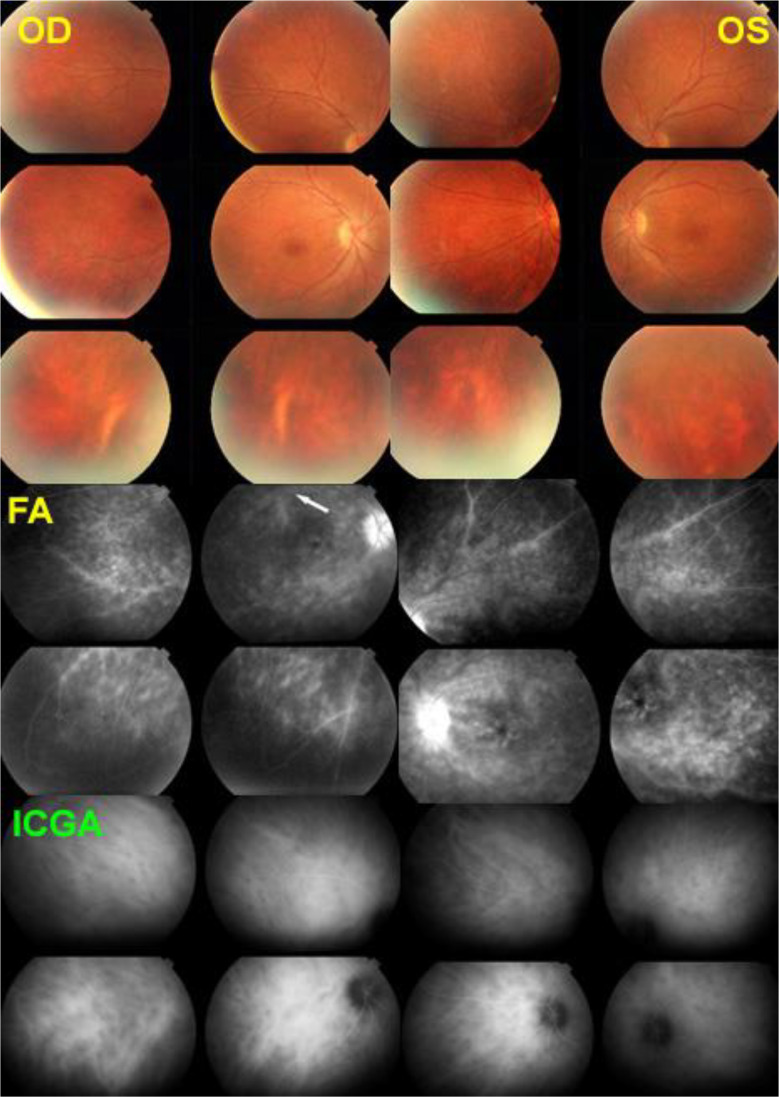
BRC retinal vasculitis, an early manifestation of the disease that can lead to the early diagnosis and prevent the BRC fundus lesions. This patient consulted for fuzzy dim vision. The fundus did not show depigmented BRC lesions. FA (middle 4 frames) was characterized by very leaky vasculitis with profuse exudation, macular edema, and thick sheathing/staining of large posterior pole vessels compatible with BRC retinal vasculitis. ICGA was performed (bottom 4 frames) and revealed numerous HDDs bilaterally. Triple immunosuppressive treatment progressively brought both the retinal and the choroidal compartment under control. The typical rice-shaped depigmented BRC fundus never appeared after 15 years of follow-up

All 21 patients in our series presented retinal involvement in the form of retinal vasculitis. In our series, 5 patients (24%) showed no clinical BRC choroidal involvement (no “birdshot lesions”) at presentation, with choroidal involvement only diagnosed by the detection of HDDs on ICGA. This indicates that retinal vasculitis appears early in the disease, sometimes in the absence of choroidal lesions, and, in case of a specific pattern, can lead the clinician to perform ICGA and search for the presence of the HLA-A29 antigen, allowing early BRC diagnosis. These early diagnosed and early treated patients never developed choroidal “birdshot lesions” [18, 19] (Fig. 1). Therefore, we were interested in finding out whether it was possible to identify a particular pattern of vasculitis that could be strongly suggestive of BRC.

The severity of retinal involvement was calculated by establishing a mean angiographic score, using the ASUWOG scoring system [17]. The retinal angiographic score at presentation amounted to 14.49 ± 5.1. Comparatively, the ICGA angiographic score was 14.29 ± 3.6, indicating that retinal and choroidal inflammation were equivalent. When comparing the retinal score of BRC to sarcoidosis and tubercular chorioretinitis, as established in previous studies using the same methodology, it was much higher than in both these entities, for which it amounted to 7.3 ± 4.6 for the former and 6.97 ± 5.08 for the latter, whereas choroidal involvement was similar, 14.2 ± 5.1 for sarcoidosis and 13.5 ± 7.06 for tubercular chorioretinitis [20, 21]. However, when comparing scores to VKH patients, also obtained in previous studies using the same methodology, the values were substantially different, with a very low retinal score of 4.06 ± 1.87 and the comparative highest choroidal score of 25.75 ± 3.88 for VKH [22]. The comparisons among these different entities show that retinal involvement is most prominent in BRC, whereas choroidal involvement is highest and most severe in VKH patients.

The type of retinal involvement had several specific angiographic characteristics, with a pattern potentially directing the clinician towards the diagnosis of BRC. The five main aspects of BRC retinal vasculitis are detailed hereafter.

**Fig. 2:**
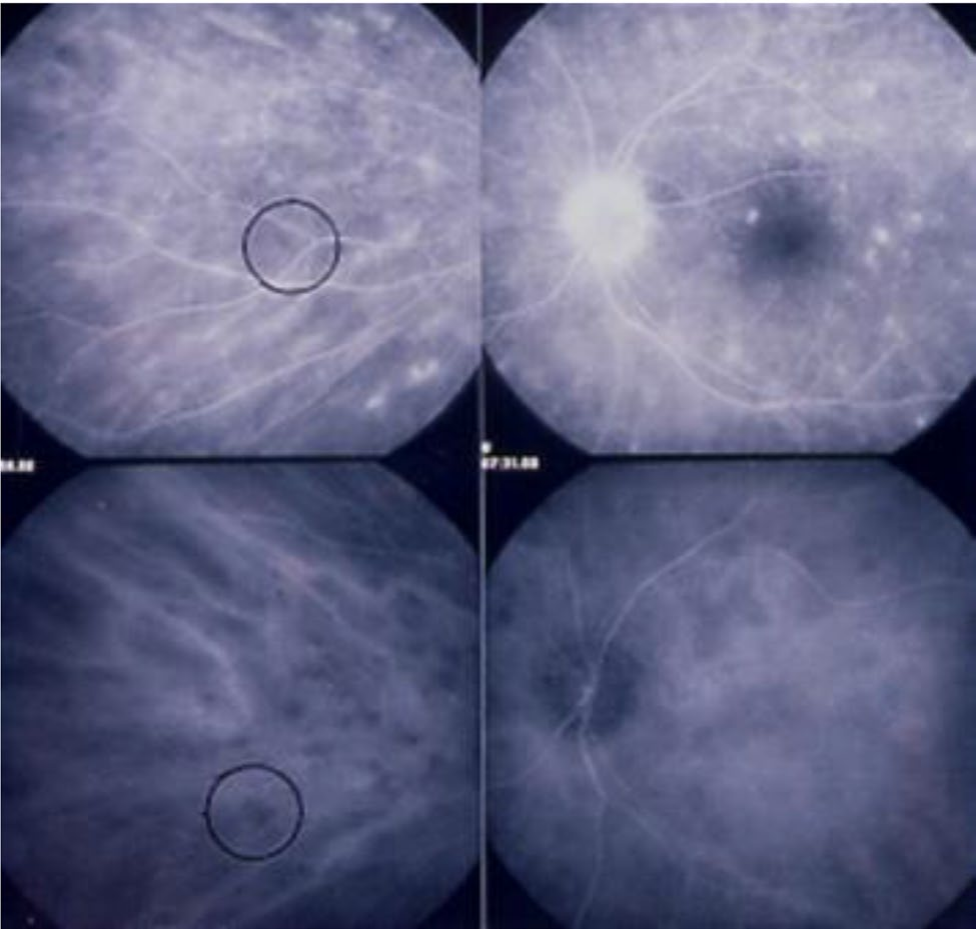
Retinal inflammation and choroidal inflammation are dual independent inflammatory events. The HDD on ICGA encircled in the bottom left frame does not correspond to the FA hyperfluorescence that is visible in the same spot (top left frame). Note that the pronounced disc hyperfluorescence on FA (top right frame) does not correspond to ICGA hyperfluorescence (bottom right frame). This latter finding indicates that inflammation is not hyperacute but more insidious in birdshot retinochoroiditis, in contrast to Vogt-Koyanagi-Harada disease, for which ICGA disc hyperfluorescence is often observed indicating very severe choroidal inflammation

Although sometimes asymmetric, retinal vasculitis was bilateral in all patients. Retinal and choroidal involvement were independent from each other, meaning that the involvement in one compartment was not the consequence of inflammation of the other compartment and vice versa, as indicated previously [23] (Fig. 2).

**Fig. 3:**
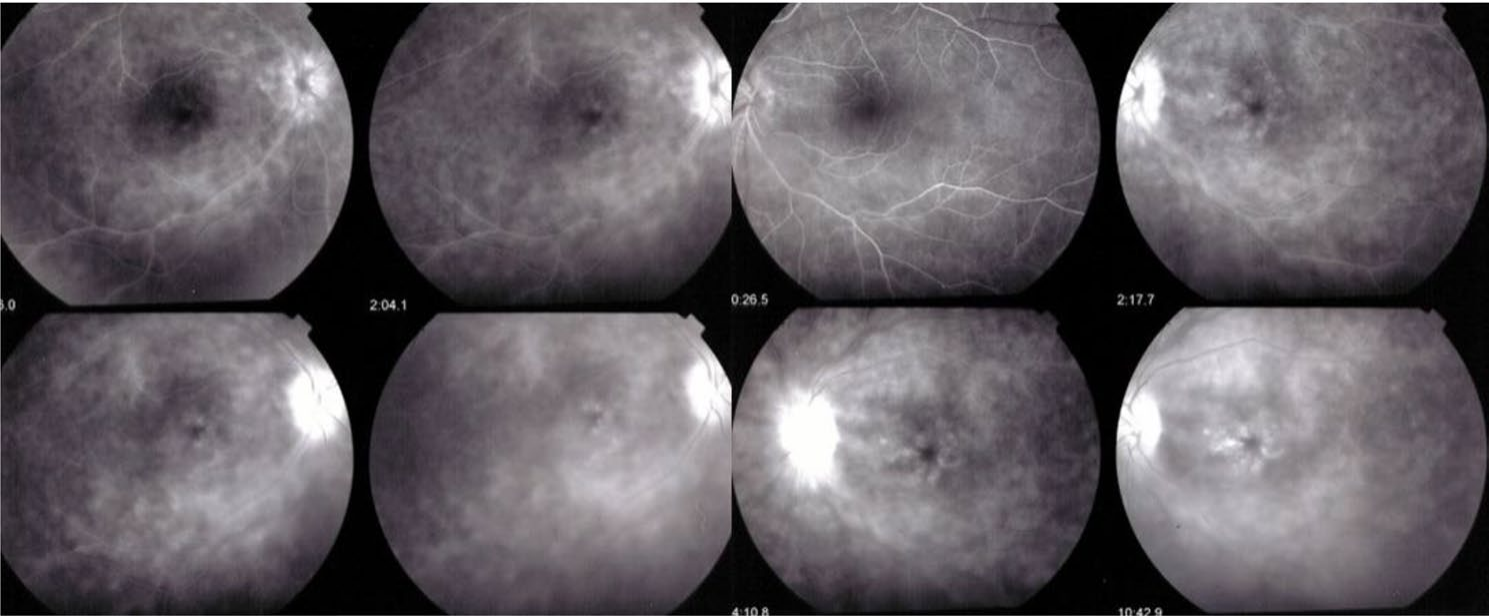
Profuse exudation with CMO. Example of a case of leaky vasculitis with profuse exudation and macular oedema that was noted in 17 cases. In this case it produced bilateral cystoid macular oedema while in many cases there was sparing of the fovea (see Fig. 4). Note on the right side, sheathing/staining of large vessels of the temporal arcades. Note also the pronounced hyperfluorescence of the discs

**Fig. 4:**
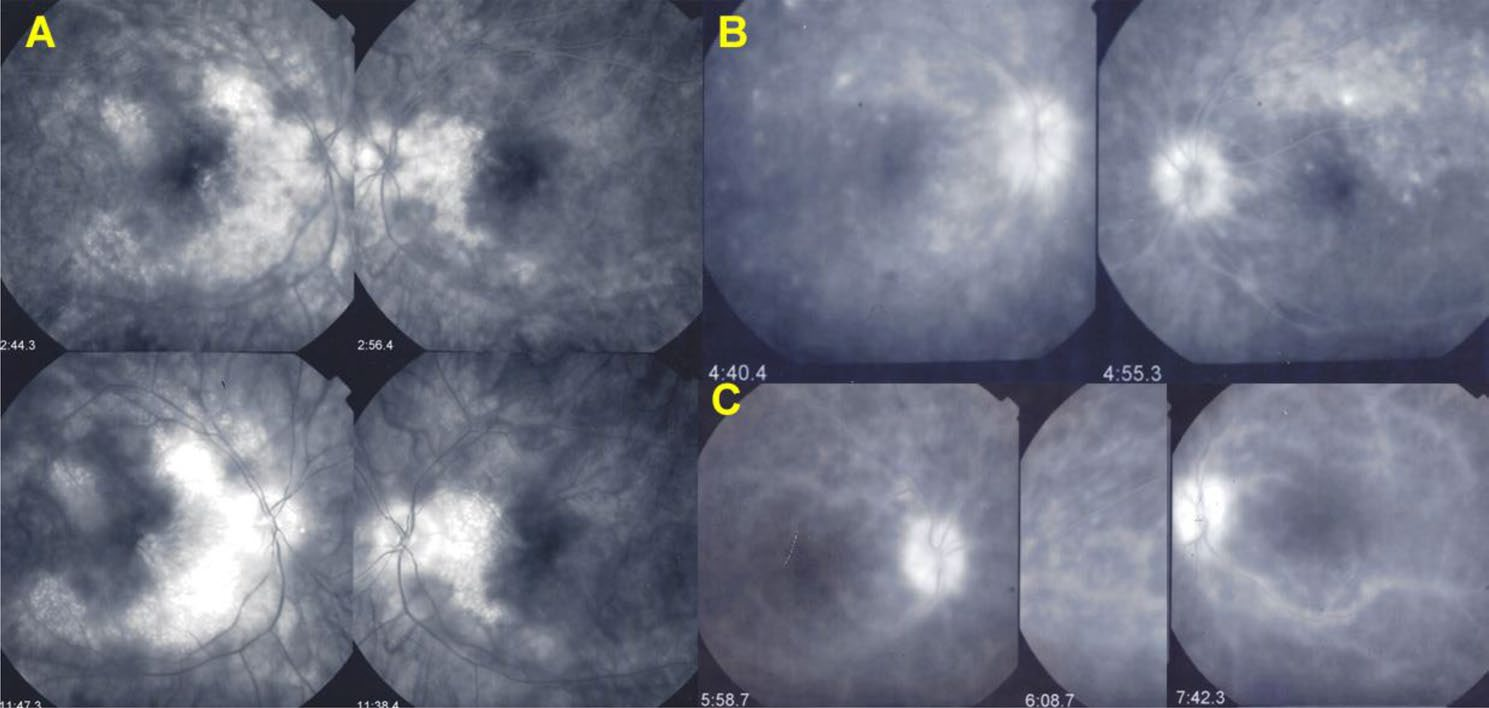
Foveal sparing of macular oedema. Three examples (**A**, **B**, **C**) of foveal sparing by the macular oedema avoiding the fovea despite prominent exudation; this is especially obvious in example **A**. Note also the staining/sheathing of large vessels of the arcades, especially apparent in example **C**

**Fig. 5:**
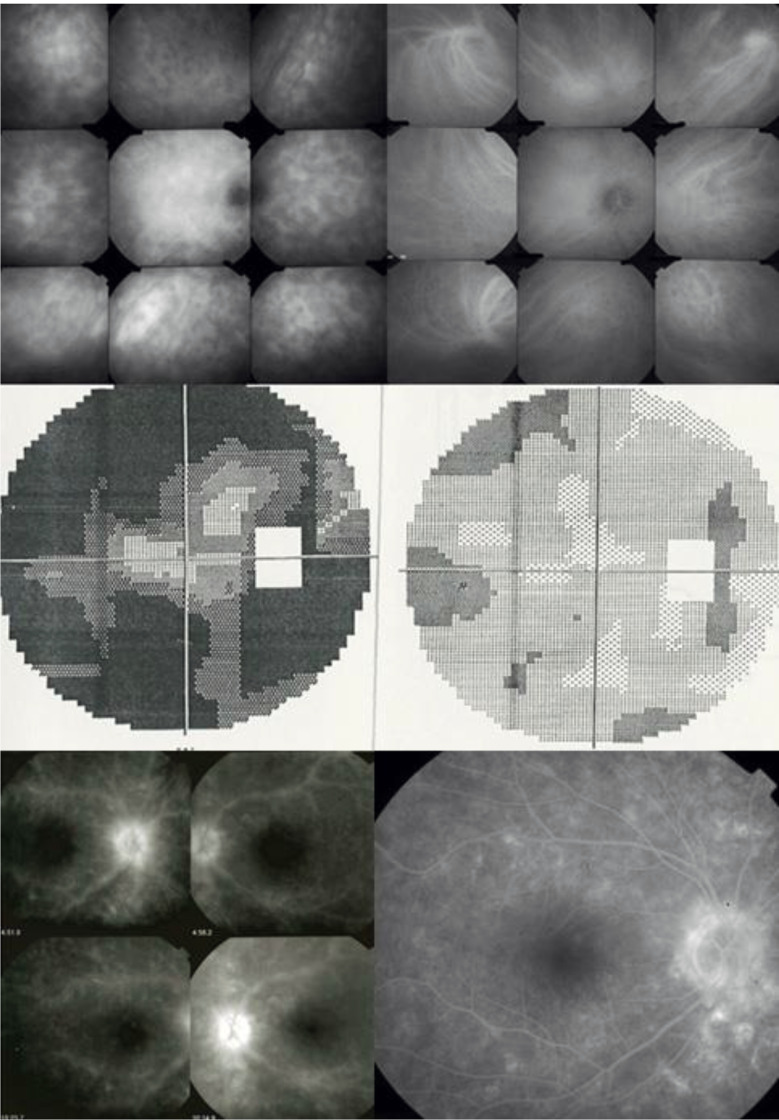
Profuse leaky vasculitis often sparing the fovea impacts visual fields but not VA. BRC at presentation with manifest choroidal involvement (top left 9 frames with numerous HDDs). Pronounced retinal vasculitis and disc hyperfluorescence (bottom left). Visual field testing showed severe impairment with tubular visual fields due to severe retinal involvement, while VA remained full because of foveal sparing. After treatment, retinal involvement recovered (bottom right) with almost complete normalisation of the visual field (middle right) and the choroiditis (top right)

Firstly, retinal involvement was characterised by a vasculitis with profuse retinal leakage in 17/21 (81%) cases. (Fig. 3)(2) Macular oedema (MO) was present in 17 patients, a consequence of profuse leakage. (Fig. 3). (3) Interestingly in 14/17 (82%) cases of MO there was sparing of the fovea. (Fig. 4) This probably explains why BRC patients very often retain a good visual acuity despite substantial involvement, while visual field defects can be pronounced (Fig. 5).

**Fig. 6:**
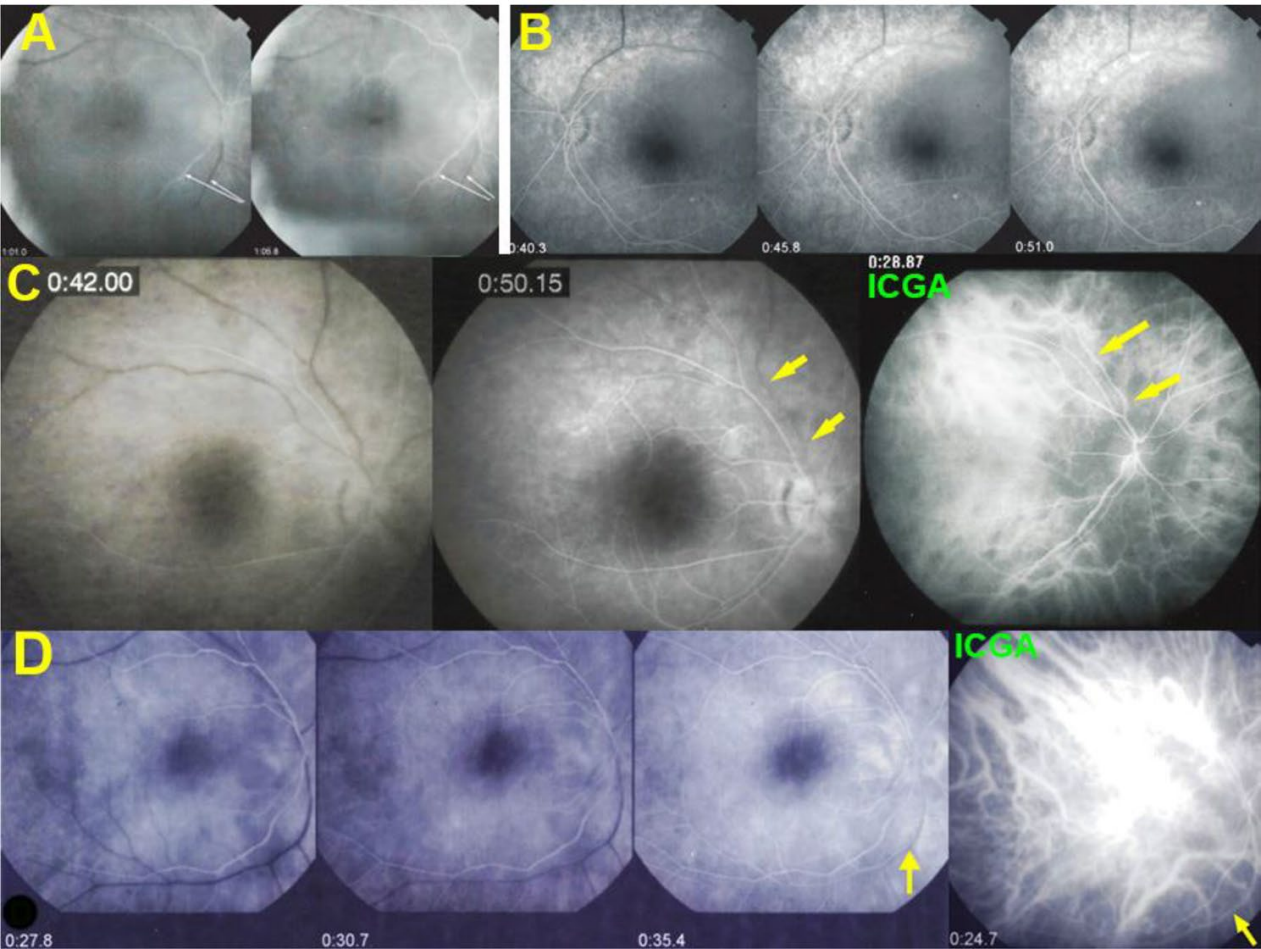
Pseudo-delay of arterio-venous retinal circulation. Four examples (**A**, **B**, **C**, **D**) of pseudo-delay of arterio-venous circulation. (**A**) shows that the pseudo-delay is > 1’05’’, a time at which some large veins are still not visible on FA (white arrows). (**B**) (3 top right frames) shows a pseudo-delay of 51 s. (**C**) shows a pseudo-delay of 50.15’’ at which time some veins are still not filled with fluorescein (yellow arrows). However, the extreme right ICGA frame shows that at 28.87’’ this very vessel is perfused (yellow arrows on extreme right ICGA frame). Example (**D**) shows a lack of marking of some veins at 35.4’’ (yellow arrow), while the extreme right ICGA frame shows that this vessel is indeed perfused at 24.7’’ (yellow arrow)

**Fig. 7:**
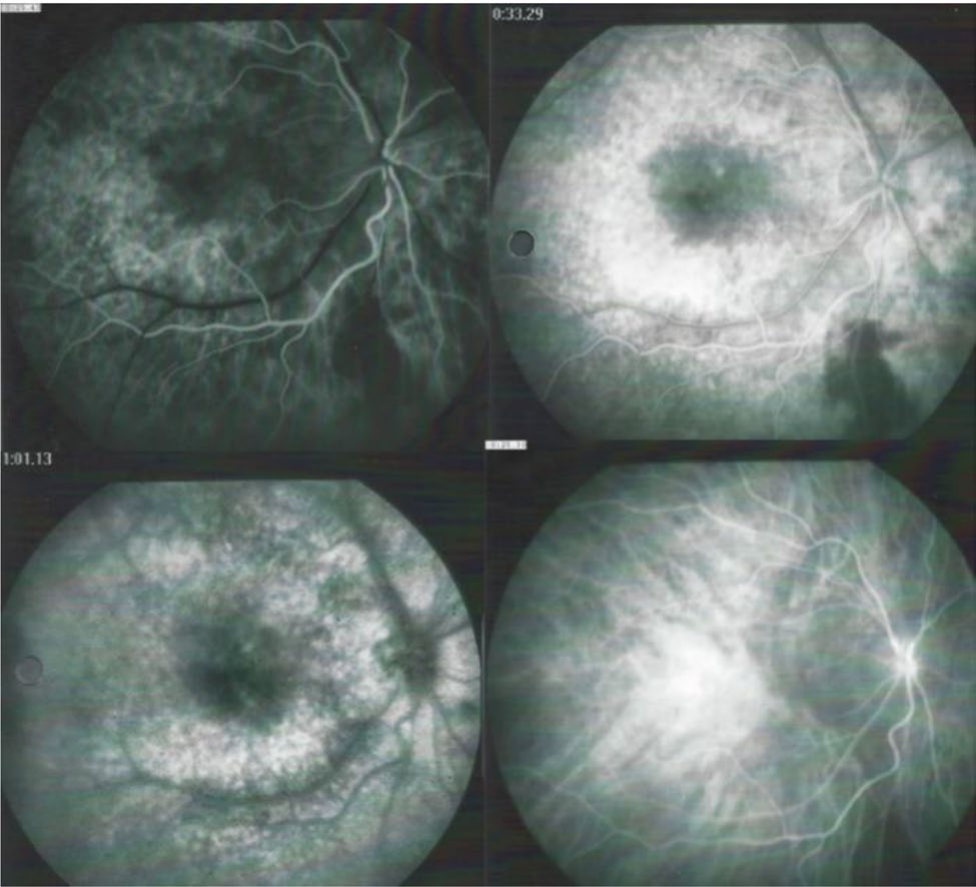
Pseudo-delay of arterio-venous retinal circulation. This BRC patient presented an extreme pseudo-delay, the veins never being marked (bottom left frame). The ICGA bottom right frame shows that the vessel is indeed perfused at 25:30’’ and that there is no real perfusion delay. The explanation for this extreme pseudo-delay is the massive intra-retinal exudation of the small fluorescein molecule, so that there is not enough dye to mark the large veins. The ICG dye which is linked to proteins has a much larger size and is not leaking from retinal vessels, indicating a normal arteriovenous circulation time

**Fig. 8:**
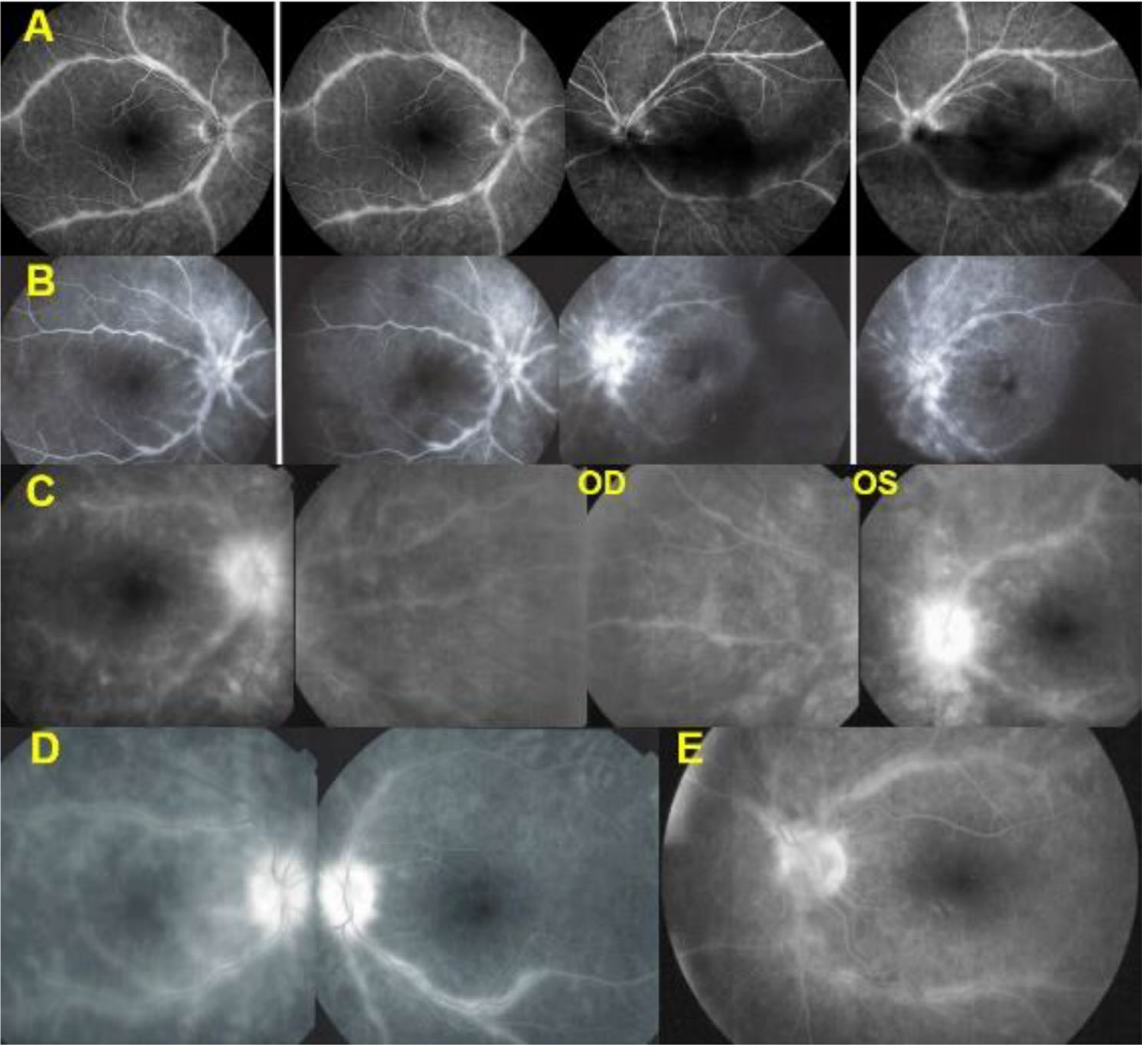
Thick sheathing/staining of large posterior vessels. **A**, **B**, **C**, **D** and **E** are examples of sheathing/staining of large vessels of the arcades. (**A**) shows wide-spread extended involvement. (**B**) is associated with left pronounced disc hyperfluorescence. In (**C**) sheathing/staining is associated with bilateral profuse disc hyperfluorescence. (**D**) and (**E**) are other examples of this typical pattern of sheathing-staining of large posterior vessels present in 13/21 patients

(4) Another consequence of the very leaky vasculitis was the delay with which the veins were marked by fluorescein a finding that was already described by Gass but was erroneously interpreted as an arterio-venous circulation delay [9]. In fact, the arterio-venous transit time was found to be normal when analysed by ICGA (Fig. 6). The “pseudo-delay” is explained by the massive exudation into the retina of the small fluorescein molecule (Fig. 7), whereas the large ICG molecular complex is remaining within the retinal vessels attesting for a normal retinal arterio-venous circulation time [10]. This circulation “ pseudo-delay” was present in 17/21 (81%) patients and the mean pseudo-circulation delay (time to venous FA marking) was 46.25 ± 22 s. In some cases, the exudation was such that the large veins were never marked (Fig. 7). For these patients 60 s was the time used for the calculation of the mean “pseudo-delay” time.

(5) Another angiographic feature seen in 13/21 (62%) patients was thick sheathing/staining of the large vessels of the vascular arcades, characterising BRC vasculitis (Fig. 8).

**Fig. 9:**
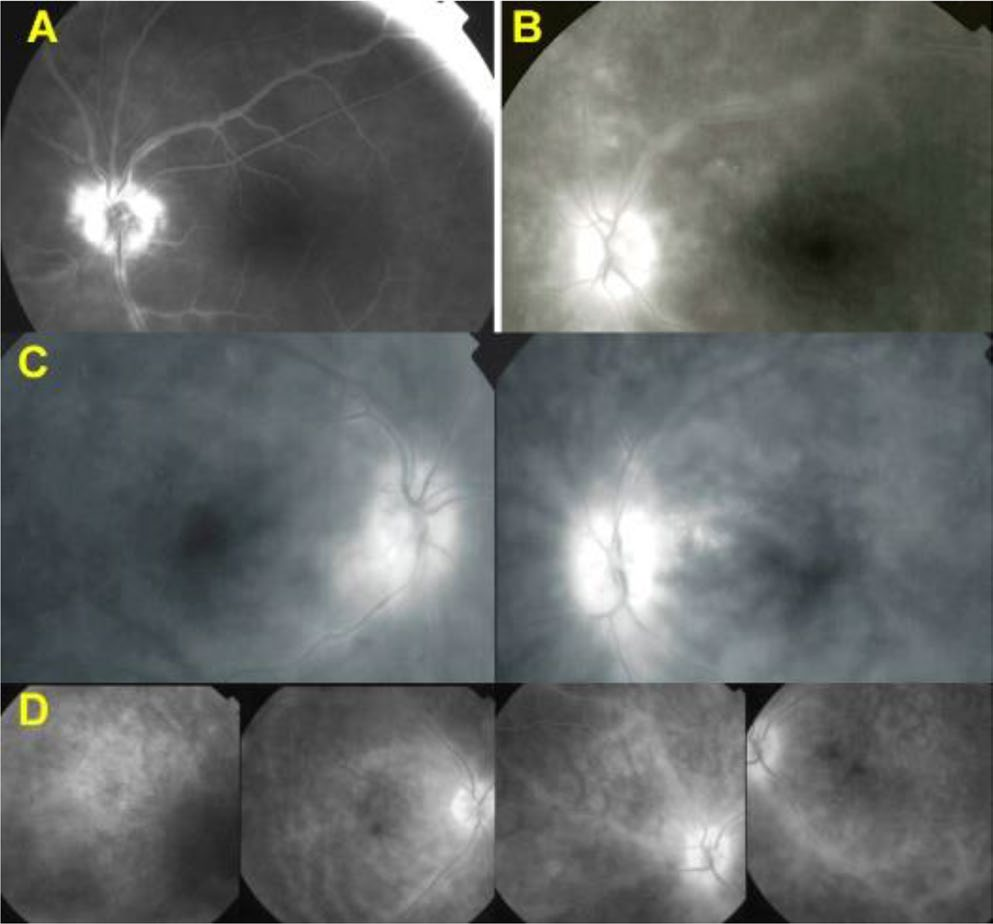
Disc hyperfluorescence. **A**, **B**, **C**, **D**. are examples of profuse disc oedema found in 86% of patients

In addition to the particular pattern described hereabove, disc hyperfluorescence was present in all patients bilaterally and was very pronounced in 36/42 (86%) of eyes with values of 3 + and over **(**Fig. 9**).**

### Other features

**Fig. 10:**
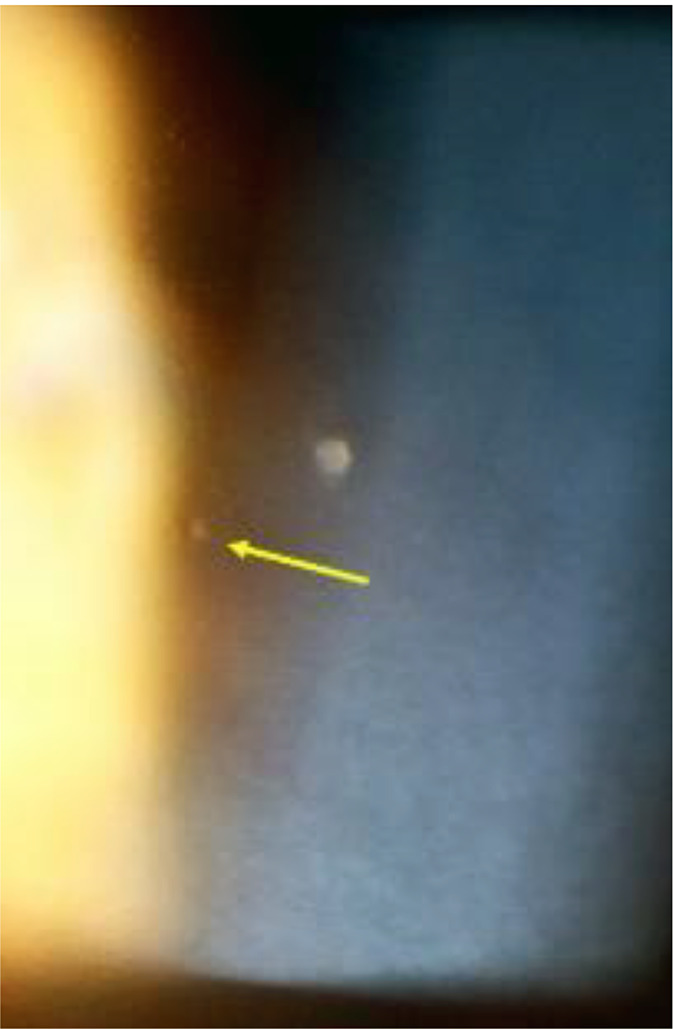
Granulomatous KPs. Two KPs, one small (arrow) and a large one as shown were found in 19% of patients, all treatment naïve patients

Anterior chamber inflammation was minimal amounting to a mean flare measured by laser flare photometry of 9.27 ± 4.77 photons/ milliseconds (ph/ms) (normal values = 4–6 ph/ms). Small mutton-fat endothelial keratic precipitates (KPs) were seen in 4/21(19%) patients indicating that BRC is a granulomatous uveitis and that KPs cannot be an exclusion criterion as listed by an international consensus workshop [2]. We had reported this rectification previously, indicating that all these patients were treatment naïve [24] (Fig. 10).

### ICGA features

**Fig. 11:**
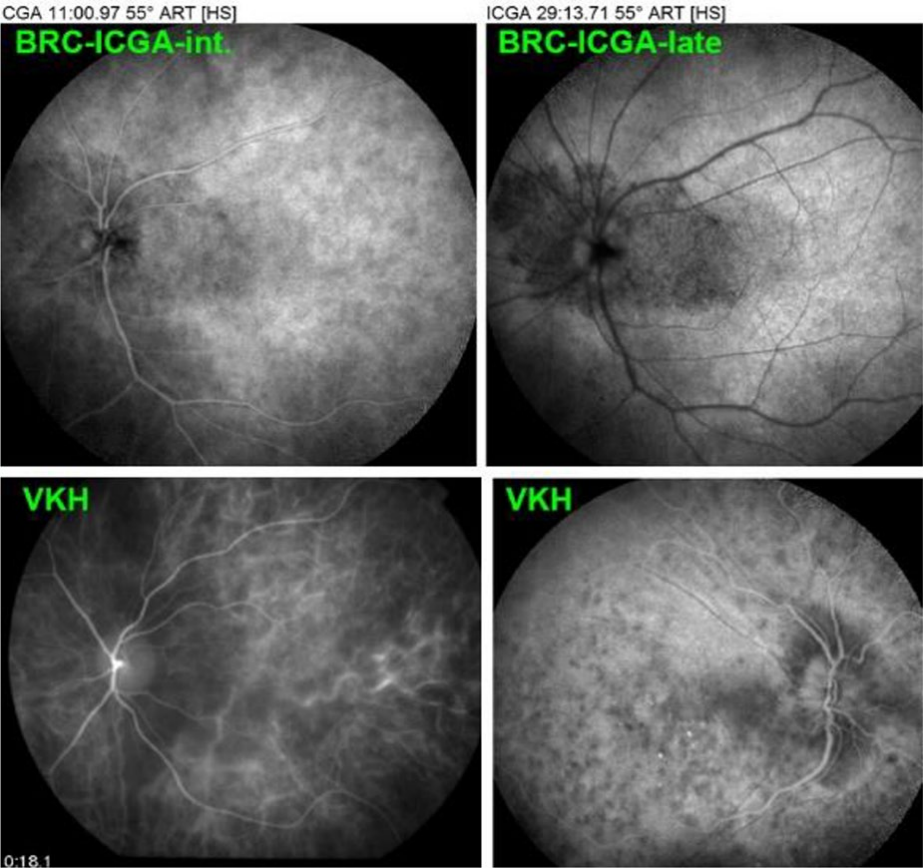
ICGA features. The two main ICGA features of HDDs and fuzziness of choroidal vessels were present in all patients (top left frame). However, HDDs tended to become isofluorescent on late angiographic frames (top right frame) in some patients. In none of the patients were there signs of hyperacute inflammation such as early hyperfluorescent vessels (bottom) or ICGA disc hyperfluorescence (bottom right) as they are found in VKH disease, a hyperacute choroiditis

Although it was not the topic of this work, ICGA features were examined. As reported hereabove, the ICGA angiographic score of 14.29 indicated that choroidal involvement was comparable to other chorioretinitis entities and was of moderate severity. The usual signs of stromal choroiditis were found such as HDDs in all patients which however became isofluorescent in the late angiographic phase in some patients indicating that the choroidal foci in BRC can be of partial thickness. Fuzzy vessels were also present in all cases. However, hyperacute signs of severe choroidal inflammation such as early hyperfluorescent vessels or ICGA hyperfluorescence of the disc, as seen in VKH, were present in none of the patients (Fig. 11).

### Vitritis

**Fig. 12:**
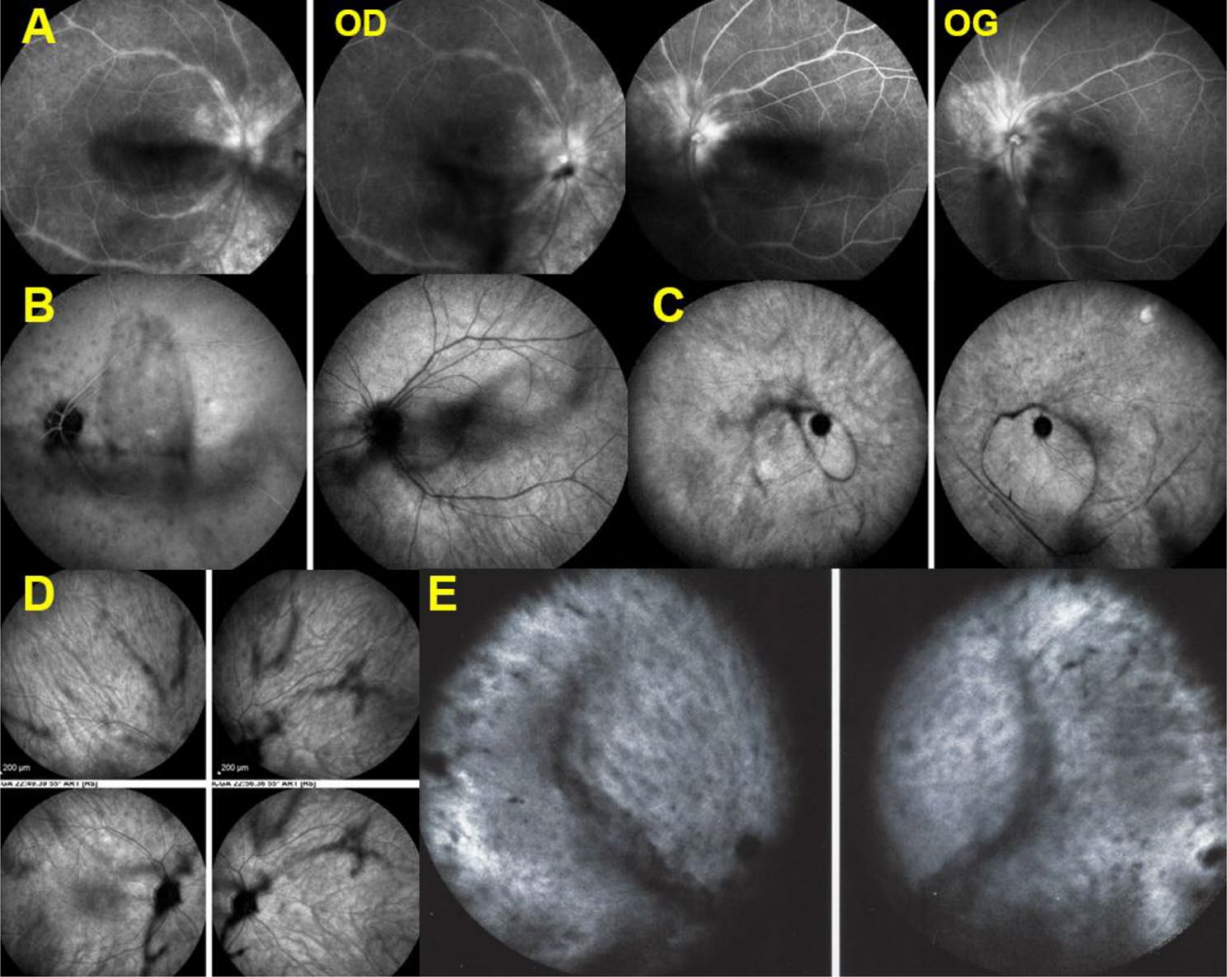
Vitritis with vitreous strands. The pattern of vitritis with vitreous strands as shown in examples **A**, **B**, **C**, **D**, **E** was a characteristic pattern present in 37% of patients

Vitritis was present in all patients bilaterally taking the aspect of large vitreous strands in 7/19 (37%) patients, constituting another characteristic albeit less frequent feature of BRC. (Fig. 12) Two patients were excluded of this calculation as they had undergone vitrectomy.

### Functional evaluation at presentation.

**Visual acuity** was relatively preserved at presentation amounting to 0.86 ± 021 (Snellen decimal values), whereas all patients showed visual field defects at presentation going from slight impairment to severe visual field defects with tubular visual fields in some instances **(**Fig. 5). The mean “mean defect” (MD) was 7.6 ± 5.3.

### Angiographic response to treatment

**Fig. 13:**
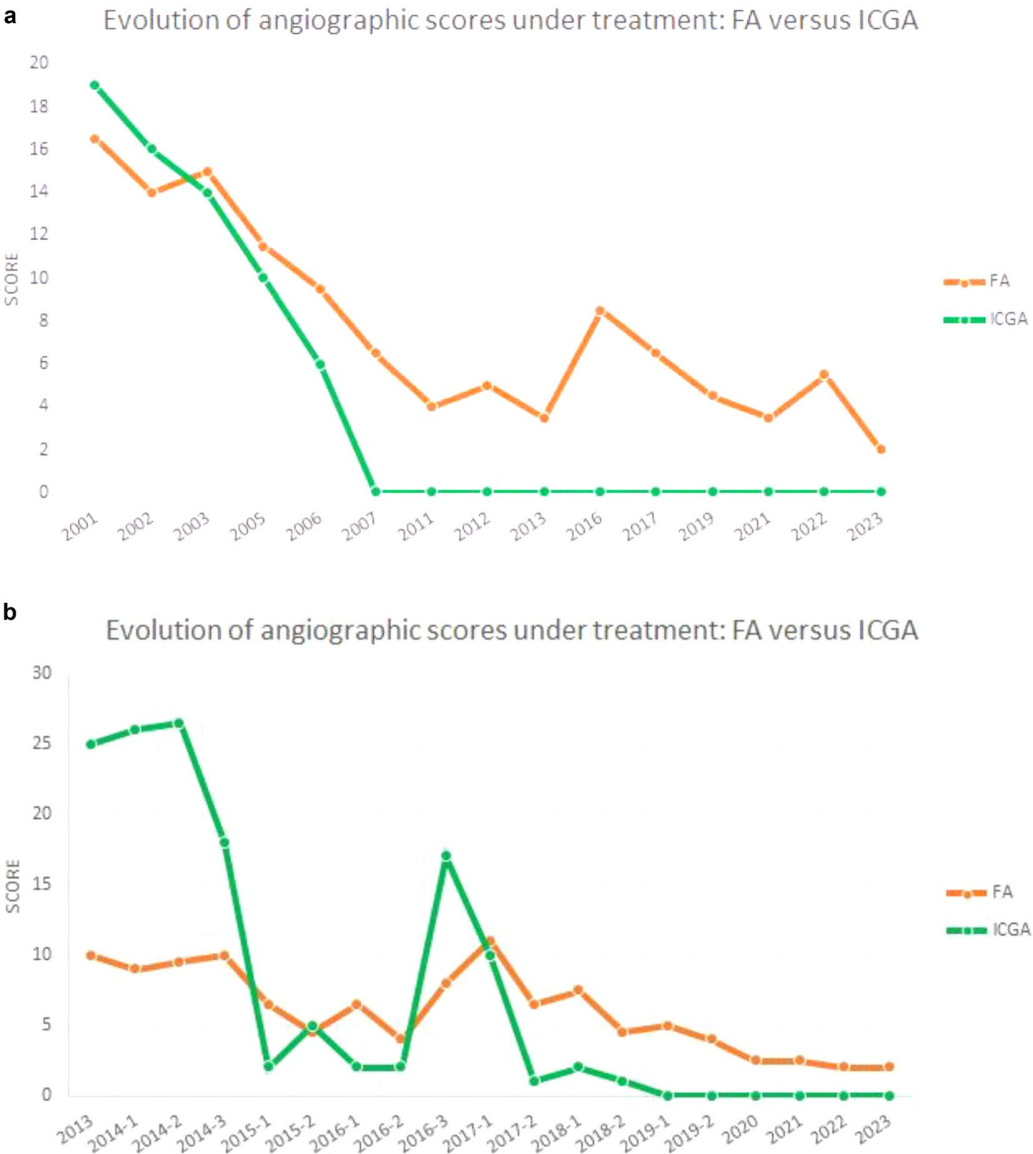
(**a**) Response to treatment of retinal versus choroidal involvement in a patient. FA/ICGA angiographic scores (average between both eyes) show the prompt response to treatment of choroiditis, while there is a slower delayed response of retinal involvement. (**b**) Response to treatment of retinal versus choroidal involvement in a patient. Another illustration of comparatively swifter response of the ICGA score (choroiditis) versus retinal vasculitis (FA score). The rebound score increase in the middle of the graph is explained by the attempt to reduce treatment that had to be reinstalled. Note that rebound inflammation is more pronounced in the choroid than retina and response to treatment increase is more rapid and complete in the choroid

For seven patients, follow-up values for the retinal (FA) and choroidal (ICGA) angiographic scores were available after a mean of 4.5 ± 1.2 years of treatment. There was a significant reduction of angiographic scores both for the retina from 14.85 ± 6.2 to 5.92 ± 3.5 (*p* < 0.009, Student’s t-test) and for the choroid from 15.42 ± 3.1 to 6.78 ± 1.7 (*p* < 0.0001), indicating that, in this series, the response to treatment was equivalent for the retinal and the choroidal compartments. However, choroiditis, as measured by ICGA score, tended to respond more quickly than retinal involvement to immunosuppressive therapy as shown by the long-term follow-up when available (Fig. 13a and b). This evolutionary pattern showing quicker response of the choroidal involvement has been reported previously by our group [23].

**Fig. 14:**
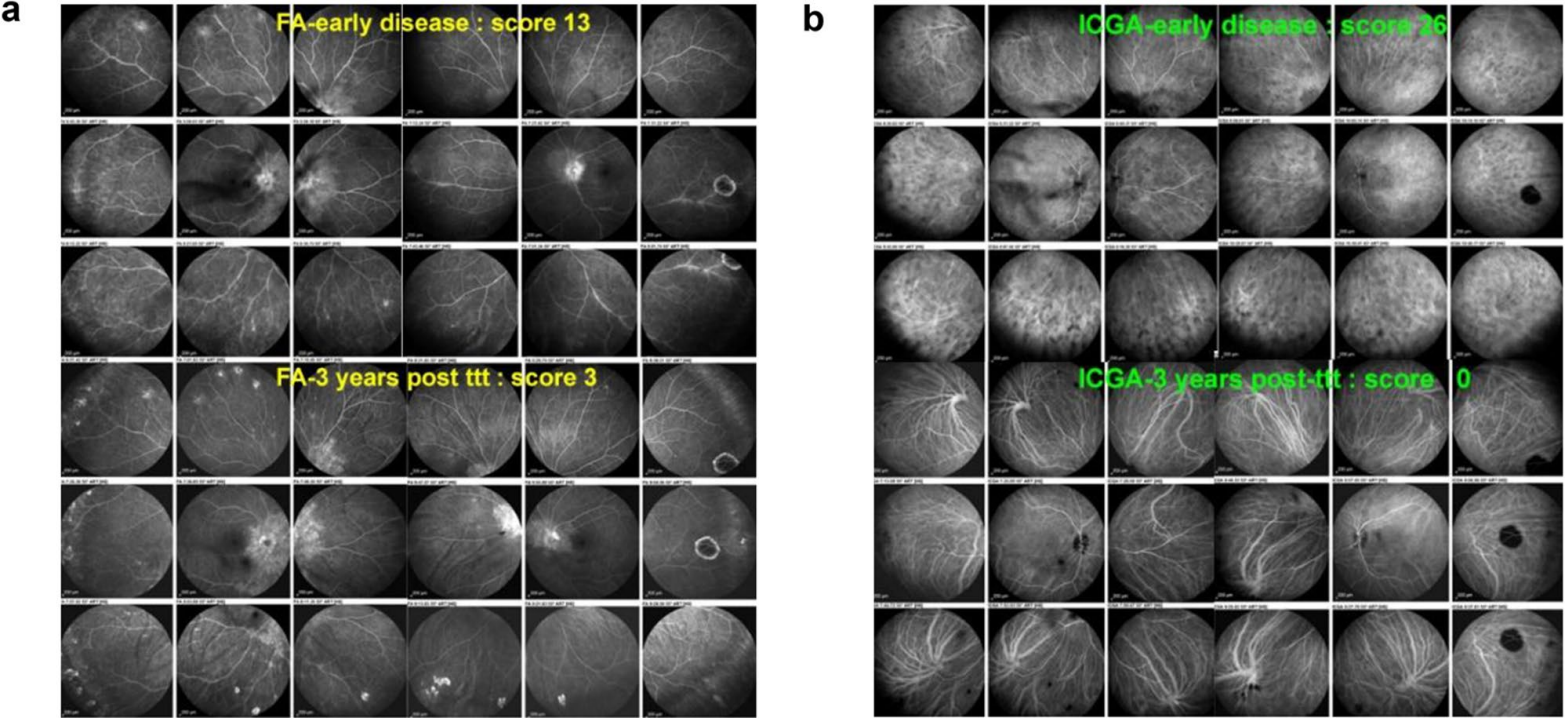
(**a**) Response to treatment of retinal involvement. This patient had minimal retinal involvement with a score of 13, having responded well to inflammation suppressive treatment after 3 years. (**b**) Response to treatment of choroidal involvement. Same patient as 13a. The extensive choroidal involvement responded well to inflammation suppressive treatment with a reduction of the ICGA score from 26 to 0 within a 3-year period

Among the patients, the respective retinal versus choroidal inflammation could be variable from one patient to another (Fig. 14a and b).

In summary, retinal involvement was present in 100% of patients characterised by a very leaky vasculitis of small and large vessels with profuse exudation, macular oedema often sparing the fovea, thick sheathing/staining of large posterior pole vessels, arterio-venous circulatory pseudo-delay, pronounced disc hyperfluorescence and thick vitreous strands. FA angiographic signs responded relatively well to inflammation suppressive treatment, although in a much slower mode when compared to the choroidal ICGA response.

## Discussion

BRC is often defined by its characteristic cream-coloured, rice-shaped “birdshot lesions”. However, such lesions correspond to choroidal (mid-stromal) scars and, by definition, are absent at the beginning of the disease and do not represent activity of disease. They are the sign of chronically evolving disease and cannot be used for early diagnosis nor for monitoring the follow-up of disease [25]. Other features, key to understanding the course of this peculiar eye inflammation have, therefore, to be taken into account. The important role of ICGA has been well established, showing HDDs [26]. ICGA is able to detect early (choroidal) HDD lesions (not to be confused with fundus “birdshot lesions”) but is usually not routinely performed in many parts of the world and so the diagnosis of BRC is often missed at this stage [4]. In the pre-ICGA era, retinal disease, analysed by FA, together with choroidal fundus “birdshot lesions” was the classical description and hallmark of the disease but represented in fact already an advanced stage of disease as indicated hereabove [2]. In the early as well as subsequent reports, FA described the retinal involvement without a systematic characterisation, being progressively neglected and remaining in the background compared to the importance given to the fundus “birdshot lesions”. In the diagnostic criteria of BRC, now obsolete, following an American birdshot workshop, retinal vasculitis was merely considered as a supporting criterion, “symptomatic” of the secondary role attributed to retinal vasculitis, while it is a crucial early manifestation that can orient the clinician towards the diagnosis of BRC [2] Table 1.

**Table 1:** BRC retinal vasculitis pattern

Retinal vasculitis	21/21	100%
Specific angiographic features		
1. Profuse retinal leakage	17/21	81%
2. Macular oedema	17/21	81%
3. Foveal sparing	14/17	82%
4. Retinal arterio- circulatory "pseudo-delay"	17/21	81%
5. Thick sheating/staining of large posterior vessels	13/21	68%
Additional less specific but helpul features		
Profuse disc hyperfluorescence	21/21	100%
Thick vitreous strands	7/19	37%

Our purpose here was to give retinal vasculitis the preponderant place it deserves as the early manifestation of BRC and systematically define the characteristics and the diagnostic usefulness of the retinal component of the disease.

In our study, we have identified key retinal features including profuse vascular leakage, macular oedema often sparing of the fovea, retinal arterio-venous circulatory “pseudo-delay”, thick sheathing/staining of large posterior pole vessels and pronounced disc hyperfluorescence.

Profuse vascular leakage and pronounced disc hyperfluorescence could correspond to a sensitive parameter, however not very specific, if sensitivity/specificity calculations were performed. Indeed, they were present and bilateral in all our patients. Optic disc oedema has been reported recently as a prominent feature of BRC, confirming our proper findings [27].

A further characteristic pattern of BRC retinitis was the fact that in over 80% of macular oedema cases the fovea was spared. This probably explains why visual acuity is mostly preserved in BRC patients, while the visual field is altered, sometimes severely, in all patients at presentation due to profuse intraretinal leakage [28]. Profuse retinal leakage was also identified by OCT, showing extensive thickening in the acute exudative phase [13].

Among the more specific features of BRC retinal vasculitis was the retinal arterio-venous circulatory “pseudo-delay” present in over 80% of cases, which is explained by the massive leakage of large and small vessels into the retina with the consequence that there is not sufficient fluorescein reaching the veins with delayed or absent marking on FA. If ICGA arterio-venous circulation time is considered, the transit time is however normal as the circulating large ICG-protein molecular complex is not extravasating from retinal vessels [10]. This phenomenon of circulatory delay was described by Gass in his article on vitiliginous chorioretinitis and was erroneously considered as a true perfusion delay as ICGA was not available at that time [9].

Another relatively specific feature was the thick sheathing/staining of large vessels of the posterior arcades merging into the disc. This angiographic finding, termed as “contiguous, perineural vascular leakage” in a recent report was found to be charcteristic for BRC [29]. This pattern was found in 57.8% of BRC patients in the reported study which corresponds to the proportion of 62% of cases that we identified in our series.

Finally, the presence of large vitreous strands, noted in more than one third of cases, despite being unspecific, was considered an additional feature that can contribute, if present, towards suspecting BRC retinal vasculitis.

Taken together, these FA features, present in early disease, often before fundus choroidal “birdshot lesions” are identified, are of utmost importance to the clinician as they represent a very strong diagnostic constellation for BRC, inciting him to perform ICGA in search of HDDs and confirm the diagnosis by searching the presence of the HLA-A29 histocompatibility antigen. Indeed, thick sheathing/staining of vessels of the posterior vascular arcades was even found to have a positive predictive value for BRC [29].

We identified another factor that could prove to be helpful in orienting the clinician towards BRC retinal vasculitis. Indeed, the retinal angiographic score was high when compared to sarcoidosis or tubercular chorioretinitis or VKH, as evidenced in previous studies performed with the same methodology. The retinal to choroidal angiographic score ratio was highest in BRC and lowest in VKH indicating that BRC had the most severe retinal vasculitis among the 4 different entities considered and VKH the least severe retinal involvement. Retinal and choroidal involvements were found to be equally prominent in BRC.

Except in the study cited hereabove [29], retinal vasculitis has been described in many reports on BRC but has never been considered comprehensively as the primary and exclusive focus of analysis.

According to our findings, the main diagnostic steps for BRC are to identify retinal vasculitis with a pattern compatible with BRC, as put forward in our study, followed by the search for stromal choroiditis using ICGA and when present, perform search of the HLA-A29 antigen to confirm the diagnosis. Complementary multimodal investigations, including optical coherence tomography (OCT), visual field testing and microperimetry are subsequently useful for the follow-up of the disease in addition to FA and ICGA [13–16].

As far as response to treatment was concerned, retinal involvement was slower to respond when compared to choroidal involvement that had a more rapid reduction of the angiographic score, a finding that we have reported previously [23]. This was also reported by Pohlmann et al., stating in their study a persistence of vascular leakage and disc hyperfluorescence in respectively 55% and 82% of their cases despite immunomodulatory treatment. Interestingly they also observed less choroidal lesions with only 23% of the cases presenting HDDs [30].

Our findings add substantial elements to be considered in the diagnosis of BRC. The diagnostic criteria of BRC published in 2006 [2] have since been revised and corrected because of the many shortcomings [31]. These diagnostic criteria were subdivided into required and supportive findings. They did not take into account ICGA, one of the most important diagnostic tests. We know by now that birdshot lesions are not required any longer for diagnosis, as diagnostic HDDs on ICGA appear well before the “birdshot lesions”. We also know that HLA-A29 is not only a supportive but a required criterium as the positivity rate is close to 100%, if the appropriate laboratory test is used. Finally retinal vasculitis was largely ignored in these diagnostic criteria and merely considered as a supportive element of BRC, illustrating the minor importance attributed to this feature in the past. As was shown in the present study, retinal vasculitis is indeed an essential component of the disease and if carefully analysed, identifying the particular features reported here, is crucial to lead the clinician to the early diagnosis. Our study further showed that KPs should not be considered as an exclusion criterion.

In conclusion, even though the pathophysiology of BRC is still not well understood, the link to HLA-A29 is clear. We still don’t know against which antigen this presumably autoimmune disease is directed. It is clear that both the choroid and retina are primary independent sites of inflammation, and both follow a distinctive evolution. Our results also indicate that retinal vasculitis is slower to respond to treatment. As indicated hereabove the presence of retinal vasculitis has been described in many reports but has rarely been analysed comprehensively per se. Our study suggests that the importance of retinal vasculitis has to be revalued in the appraisal of BRC, especially in view of the early diagnosis it can represent.

Practically, the diagnosis of BRC should be considered (1) when facing any bilateral compatible retinal vasculitis on FA showing a severe retinal vasculitis of a certain type, (2) completed by typical choroidal involvement explored by ICGA and (3) HLA-A29 positivity. Investigations should be completed by exploring visual function including visual field testing and by the exploration of retinal morphology by OCT.

## Data Availability

Refer to corresponding author.

## Abbreviations

29 (HLA-A29): Human leukocyte Antigen A
PSC: Primary stromal choroiditis
BRC: HLA-A29 birdshot retinochoroiditis
ICGA: Indocyanine green angiography
HDDs: Hypofluorescent dark dots
FA: Fluorescein angiography
OCT: Optical coherence tomography
VF: Visual field
MP: Microperimetry
OCT-A: OCT angiography
VKH: Vogt-Koyanagi-Harada
